# Zanthoxoaporphines A–C: Three new larvicidal dibenzo[*de*,*g*]quinolin-7-one alkaloids from *Zanthoxylum paracanthum* (Rutaceae)

**DOI:** 10.3762/bjoc.9.47

**Published:** 2013-02-27

**Authors:** Fidelis N Samita, Louis P Sandjo, Isaiah O Ndiege, Ahmed Hassanali, Wilber Lwande

**Affiliations:** 1 Department of Chemistry, Maseno University, P.O. Private Bag Maseno, Kenya; 2 Department of Chemistry, Kenyatta University, P.O. Box 43844, Nairobi 00100, Kenya; 3 Department of Organic chemistry, University of Yaoundé 1, P.O. Box 812 Yaoundé, Cameroon; 4 Behavioural and Chemical Ecology Department, International Centre for Insect Physiology and Ecology, P.O. Box 30772, Nairobi 00100, Kenya

**Keywords:** alkaloids, larvicidal activity, *Zanthoxylum paracanthum*

## Abstract

The bioassay-guided purification of *Zanthoxylum paracanthum* (Rutaceae) extracts led to the isolation of three new alkaloids, namely 1-hydroxy-10-methoxy-7*H*-dibenzo[*de*,*g*]quinolin-7-one (zanthoxoaporphine A, **2**), 1-hydroxy-7*H*-dibenzo[*de*,*g*]quinolin-7-one (zanthoxoaporphine B, **3**) and 1,8-dihydroxy-9-methoxy-7*H*-dibenzo[*de*,*g*]quinolin-7-one (zanthoxaporphine C, **4**), and a known lignan identified as sesamin (**1**). Isolation and purification of the constituent compounds was achieved through conventional chromatographic methods. The chemical structures of the isolated compounds were determined on the basis of UV, IR, NMR and MS data, and confirmed by comparison with those reported in the literature. The larvicidal activity of some of the isolated compounds was investigated by using third-instar *Anopheles gambiae* larvae.

## Introduction

*Zanthoxylum paracanthum* is a tree belonging to the Rutaceae family. The mature tree is at least 10 m tall. It has branches with straight or upturned spines that are 3–11 mm long and has leaves with 11–23 leaflets. It is found mainly in moist or dried forests or in closed thickets near the sea [1]. Many communities in east Africa use several plant species of this family in traditional pharmacopoeia. The bark of *Z. amaniensis* is chewed to relieve tooth ache. The leaf decoction of *Z. chalybea* is administered to children suffering from kwashiorkor and as a beverage for the treatment of oedema [2]. Although, *Z. paracanthum* is not known to be used by Kenyan traditional healers, previous studies reported species from the same genus as rich in alkaloids [3–5], which constitute the most abundant class of compounds in this genus, while coumarins [6], lignans [3] and triterpenoids, especially limonoids [7], are the minor constituents. Different classes of alkaloids have been found and reported from *Zanthoxylum* species, namely isoquinolines represented by benzophenanthridines [8], oxoaporphines [9], aporphines [10] and benzylisoquinoline [11]; and quinolines represented by quinolones [12] and furoquinolines [10]. Others include carbazoles [13], pyrido-indoles [12] and quinazolines [14]. Arylethanamides and amines have also been previously reported from this genus [15–16]. Thus, the reports of the presence of *N*-containing secondary metabolites prompted us to investigate the larvicidal effect of extracts and isolated compounds as an alternative for malaria vector control, since synthetic organic insecticides are often harmful to nontarget organisms, including human beings, and have negative environmental impacts. We herein report the bioassay-guided isolation of four compounds including three new ones and the larvicidal activity of two of them.

## Results and Discussion

Stem bark of *Z. paracanthum* was successively extracted with hexane, methylene chloride, ethyl acetate and methanol, respectively, yielding four extracts A–D. Extract B showed significant larvicidal activity (Table 1), whereas the others did not exhibit reasonable activity against third-instar *Anopheles gambiae* larvae.

**Table 1:** Lethal concentration 50 (μg/mL) of extract A, B, C, and D after 24, 48 and 72 h.

	24 h	48 h	72 h

A (Hex extract)	> 100	> 100	> 100
B (DCM extract)	3.10	2.82	2.77
C (EA extract)	> 100	> 100	> 100
D (MeOH extract)	> 100	> 100	> 100

Extract B was therefore subjected to repeated column chromatography affording 60 fractions, which were pooled into 18 subfractions (B_1_–B_18_) based on comparative TLC. B_1_, B_3_, B_5_, B_6_, B_9_–B_12_, B_15_ and B_16_ yielded substantial amounts to be tested against third-instar *Anopheles gambiae* larvae. Consequently, subfractions B_1_, B_3_, B_5_ and B_16_ did not exhibit any larvicidal activity at 10 μg/mL after 72 hours. However, subfraction B_9_–B_12_ and B_15_ displayed 30, 50, 20, 34 and 10% mortality, respectively, at 10 ppm after 72 hours. B_6_ showed 84% mortality after 72 hours at the same concentration.

**Figure 1:**
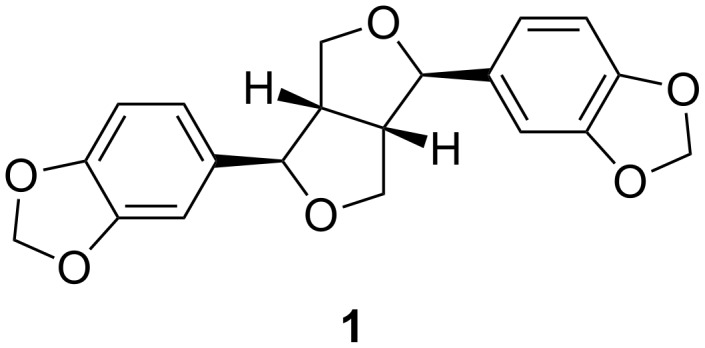
Known compound sesamin (**1**) isolated from methylene extract of stem bark of *Z. paracanthum*.

**Figure 2:**
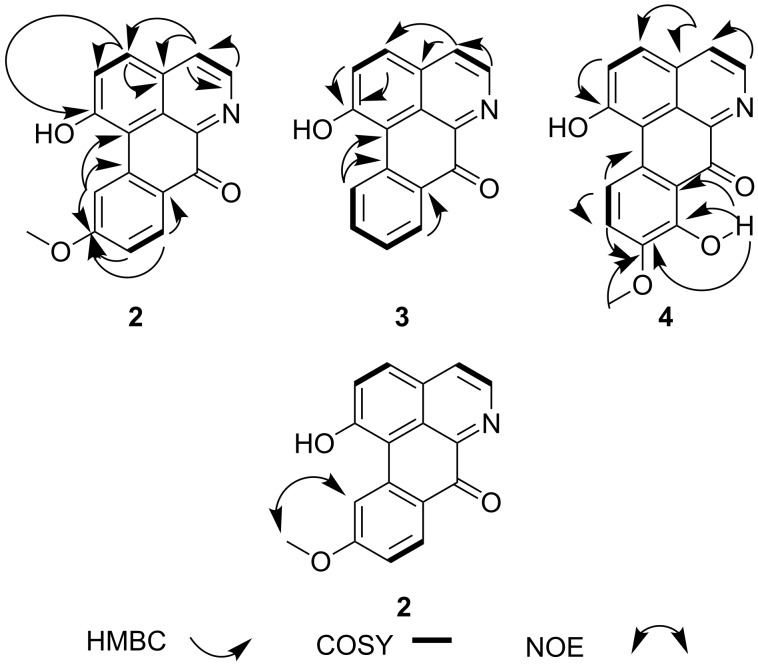
COSY, HMBC and NOE correlations of compounds **2**, **3** and **4**.

Sesamin (**1**, Figure 1) was isolated as a white solid from B_6_ and its NMR data were in agreement with those reported by Lee and Yen [17]. Zanthoxoaporphine A (**2**) was obtained as a yellowish solid from subfractions B_11_ and B_12_. Its high-resolution ESIMS showed the pseudo molecular ion peak [M + H]^+^ at *m*/*z* 278.0822 (calcd 278.0812) corresponding to the molecular formula C_17_H_11_O_3_N accounting for thirteen double bond equivalents. The UV absorption bands at λ_max_ 264, 309 and 350 nm were close to those of an oxoaporphine [18]. The IR band observed at ν_max_1653 cm^−1^ was diagnostic of a carbonyl (C=O) in a N-substituted α-iminoketone group. The ^1^H NMR spectrum of **2** (data, see Table 2) displayed four doublets of two AB patterns, one at δ 7.86 and 8.76 (*J* = 5.6 Hz) whereas the others were at δ 6.96 and 8.04 (*J* = 9.6 Hz). Three signals of an ABX-system including a doublet at δ 7.95 (*J* = 8.0 Hz), a broad doublet at δ 7.07 (*J* = 8.0 Hz), and a broad singlet at δ 8.18 were further observed; the remaining signal was attributed to a methoxy group at δ 3.98 located at C-10 since its proton had a NOE contact (Figure 2) with the hydrogen at δ 8.18. The complete assignment was achieved by using HMBC data (Figure 2), which revealed correlations between H-4 at δ 7.86 and C-5 (δ 145.0), C-3a (δ 130.2) and C-3 (δ 139.5). Besides, long-range interactions were also observed between H-2 (δ 6.96) and carbon at δ 139.5 (C-3), 159.7 (C-1) and 123.1 (C-1a), which suggested the isoquinoline core. Further correlations were noted from H-11 (δ 8.18) to C-11a (δ 141.9), C-10 (δ 163.2) and C-9 (δ 114.8). Finally, the interaction between H-8 and C-7a in conjunction with the carbonyl signal revealed in the ^13^C NMR spectrum at δ 180.1, suggested a benzocyclohexenone scaffold. Combining the isoquinoline and the benzocyclohexenone skeletons led to the 7*H*-dibenzo[*de*,*g*]quinolone-7-one scaffold. The above data were compared to those reported for fissiceine [18] leading to identification of **2** as 1-hydroxy-10-methoxy-7*H*-dibenzo[*de*,*g*]quinolone-7-one, which was assigned the trivial name zanthoxoaporphine A.

**Table 2:** NMR data of compounds **2**, **3** and **4** in CDCl_3_.

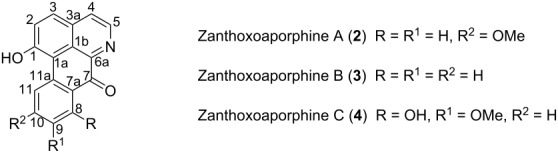						

	δ_H_			δ_C_		

Position	Compound **2**	Compound **3**	Compound **4**	Compound **2**	Compound **3**	Compound **4**

1	–	–	–	159.7	159.4	160.4
1a	–	–	–	123.1	124.3	119.4
1b	–	–	–	132.5	131.9	132.1
2	6.96 (1H, d, 9.6)	6.99 (1H, d, 9.8)	7.04 (1H, d, 9.5)	129.4	128.8	126.7
3	8.04 (1H, d, 9.6)	8.03 (1H, d, 9.8)	8.15 (1H, d, 9.5)	131.5	139.5	141.1
3a	–	–	–	134.9	130.2	130.8
4	7.86 (1H, d, 5.6)	7.97 (1H, d, 5.0)	7.87 (1H, d, 5.2)	115.9	116.4	116.1
5	8.76 (1H, d, 5.6)	8.82 (1H, d, 5.0)	8.83 (1H, d, 5.2)	145.0	145.7	146.9
6a	–	–	–	139.0	136.1	136.9
7	–	–	–	180.1	180.7	187.7
7a	–	–	–	117.2	124.5	128.3
8	7.95 (1H, d, 8.0)	8.67 (1H, d, 8.1)	–	124.0	117.2	147.7
9	7.07 (1H, br d, 8.0)	7.71 (1H, d, 7.6)	–	114.8	130.8	152.1
10	–	7.53 (1H, d, 7.6)	7.52 (1H, d, 8.6)	163.2	125.6	113.1
11	8.18 (1H, br s)	8.11 (1H, d, 8.1)	7.07 (1H, d, 8.6)	101.5	122.6	111.1
11a	–	–	–	141.9	139.3	137.2
OMe	3.98 (3H, s)	–	4.03 (3H, s)	56.0	–	56.9
OH	–	–	12.2 (1H, s)	–	–	–

Zanthoxoaporphine B (**3**) was obtained from fractions B_9_ and B_10_ as an orange powder. Its HRMS–ESI showed the pseudo molecular ion peak [M + H]^+^ at *m*/*z* 248.0715 (calcd 248.0706) corresponding to the molecular formula C_16_H_9_O_2_N and accounting for thirteen double bond equivalents. The molecular ion peak of **3** was 30 amu (atomic mass units) lower than that of **2** suggesting a loss of an oxymethylene group to give the demethoxy analogue of **2**. Similarly**,** the UV absorption bands of compound **3** at λ_max_ 253, 297 and 378 nm were close to those of a 7-oxoaporphine [18]. The IR band observed at ν_max_ 1647 cm^−1^ indicated a conjugated ketone. Similarly, the ^1^H NMR data (Table 2) revealed four doublets of two AB-systems, the first at δ 7.97 (*J* = 5.0 Hz) and 8.82 (*J* = 5.0 Hz) while the second at δ 6.99 and 8.03 (*J* = 9.8 Hz) corresponding to the proton signals of the hydroxyisoquinoline part. Moreover, four signals of an *ortho*-substituted phenyl residue were observed at δ 8.67 (d, *J* = 8.1 Hz), 7.71 (br d, *J* = 7.6 Hz), 7.53 (br d, *J* = 7.6 Hz), 8.11 (d, *J* = 8.1 Hz). The complete assignment was done by using long-range correlations from the HMBC data (Figure 2) and by comparison of the NMR data of **3** with those previously reported [19]. The HMBC spectrum revealed cross peaks between H-4 (δ 7.97) and carbon atoms C-5 (δ 145.7), C-3a (δ 130.2) and C-3 (δ 139.5); and between H-11 and carbon atoms C-11a (δ 139.3) and C-10 (δ 125.6); while H-8 was correlated with C-9 (δ 130.8) and C-7a (δ 124.5). From the above-mentioned data the structure of **3** was confirmed as 1-hydroxy-7*H*-dibenzo[*de*,*g*]quinolin-7-one, trivially named zanthoxoaporphine B.

Zanthoxoaporphine C (**4**) was obtained as an orange solid from fraction B_14_. Its molecular formula C_17_H_11_O_4_N was found on the basis of its NMR data (Table 2) and the HRMS–ESI spectrum, which showed a pseudo molecular ion peak at *m*/*z* 294.0770 (calcd 294.0761, [M + H]^+^). Compound **4** gave the same UV absorption pattern as its analogues **2** and **3** at λ_max_ 261, 320 and 403 nm, suggesting the same scaffold as the above two alkaloids. Similar to **2** and **3**, the ^1^H NMR data of compound **4** showed two AB-patterns at δ 7.87 (H-4, *J* = 5.2 Hz) and 8.83 (H-5, *J* = 5.2 Hz) and at δ 7.04 (H-2, *J* = 9.5 Hz) and 8.15 (H-3, *J* = 9.5 Hz) representing the methine groups of the isoquinoline part. The difference was found in the benzocyclohexenone part where an additional AB-pattern was observed at δ 7.52 (H-10, *J* = 8.6 Hz) and 7.07 (H-11, *J* = 8.6 Hz). The HMBC data (Figure 2) revealed interactions between H-2 and C-1 and C-2; and between H-4 and C-5, C-3a and C-3. The same contacts were further present between H-10 and C-8, C-9 and C-11 as well as between the methoxy protons at δ 4.03 and the carbon at δ 152.1. The complete spectral assignment led to the identification of compound **4** as 1,8-dihydroxy-9-methoxy-7*H*-dibenzo[*de*,*g*]quinolin-7-one, which was named zanthoxoaporphine C.

Due to the substantial amount of compounds **1** and **2,** their effect against third-instar *Anopheles gambiae* larvae was evaluated (Table 3). Compound **2** exhibited moderate larvicidal activity after 24 hours with a LC_50_ at 14.4 μg/mL, while the LC_50_ of **1** showed mild activity (LC_50_ 68.8 μg/mL). The activity of sesamin (**1**) improved significantly after 72 hours to moderate levels (LC_50_ at 10.3 μg/mL), while zanthoxoaporphine A (**2**) did not exhibit a significant change in its larvicidal activity after 3 days. Oxoaporphines and aporphines have been reported to have larvicidal activity against *A. aegypti* larvae. For example, liriodenine and (+)-dicentrine have been reported as larvicidal secondary metabolites with LD_50_ at 3.6 and 30.2 ppm after 24 hours [20–21]. Although the larvae belong to a different genus, they are from the same family, and the mode of action of these alkaloids could be the same. Zanthoxoaporphine A (**2**) and liriodenine, which are oxoaporphines, are more active than dicentrine (aporphine). The isoquinoline moiety could be the pharmacophore of this class of compounds and the presence of a ketone at C-7, C-4 or C-5 [22] seems to contribute to the activity. Sesamin, which showed moderated larvicidal activity, has also been reported to have antifeeding and growth-inhibition activities [23].

**Table 3:** Lethal concentration 50 (μg/ml) of compounds **1** and **2** after 24, 48 and 72 h.

	24 h	48 h	72 h

Sesamin (**1**)	68.8	13.0	10.3
Zanthoxoaporphine A (**2**)	14.4	12.0	11.1

## Conclusion

*Zanthoxylum* is a rich source of alkaloids with diverse biological activities. For example, oxoaporphine and aporphine analogues have shown antimalarial, antitrypanosomal [24], cytotoxic [25], antioxidant [26] and larvicidal activities [20–21]. Despite the reported bioactivities, some of the oxoaporphines and aporphines have undesirable effects, for instance liriodenine is mutagenic [27] and induces DNA damage, while corydine and atherospermidine also induce DNA damage [25]. *Zanthoxylum* is a potential source of lead compounds that can inspire the synthesis of bioactive principles based on an isoquinoline scaffold.

## Experimental

### General methods

Melting points were determined on a Sanyo Gallenkamp electronic apparatus and are uncorrected. 1D and 2D NMR spectra were carried out on a Bruker DRX-300 MHz and Bruker 600 MHz instrument, respectively. Silica gels 60H (particle size < 45 µm), GF254 and 60A (size 70–200 µm) were used to perform, respectively, flash chromatography, thin-layer chromatography, and column chromatography. Iodine–silica gel was employed as a developer to visualize the spots on the TLC plates. HRMS–EI were recorded with a JEOL JMS HX-110 mass spectrometer instrument, and EI analysis was performed by using the direct insertion probe (DIP) on a fission platform 11 mass spectrometer operating at 70 eV. IR and UV data were obtained from Shimadzu FT spectrophotometer and Beckman system Gold HPLC–UV with a diode array detector, respectively.

#### Plant material

*Z. paracanthum* (Mildbr.) Kokwaro stem bark was collected on 29th September 2001 at Mrima Hill in the Kwale district, Kenya. The plant was identified by the Herbarium in the Department of Botany, University of Nairobi where a voucher was deposited under the specimen number 2001/579.

#### Extraction and isolation

The air-dried stem bark of *Z. paracanthum* was powdered and 200 g was macerated successively with hexane (Hex), dichloromethane (DCM), ethyl acetate (EA), and methanol. The extraction process lasted 3 days with each solvent (1 L). The organic part was concentrated in vacuo to give hexane extract (A) (2 g), DCM extract (B) (6 g), EA extract (C) (3 g), and methanol extract (D) (4 g). Due to its strong larvicidal activity, extract B was purified on silica gel by using column chromatography eluting with hexane/ethyl acetate in gradient conditions to afford 60 fractions. These were pooled into 18 subfractions (B_1_–B_18_) on the basis of TLC. Compound **1** (145.0 mg) was obtained from subfraction B_6_ (eluted with Hex/EA 9:1), while compound **2** (52.4 mg) was isolated from B_11_ and B_12_ (eluted with Hex/EA 13:7). Similarly, compound **3** (2.0 mg) was obtained from B_9_ and B_10_ (eluted with Hex/EA 7:3), while B_14_ eluted with Hex/EA 1:1 yielded compound **4** (3.0 mg).

#### Characterization

Sesamin (**1**): White powder; mp 123–124 °C (lit. [28]: mp 127–129 °C).

1-Hydroxy-10-methoxy-7*H*-dibenzo[*de*,*g*]quinolone-7-one (**2**): Yellowish solid; mp 178–181 °C; IR (cm^−1^): 3628, 1662, 1653, 1576, 1279; UV (MeOH) λ_max_: 207, 264, 271, 309, 350 nm; HRMS–ESI (*m*/*z*): [M + H]^+^ calcd for 278.0812; found, 278.0822; EIMS *m*/*z*: 250 (100%), 207 (65%), 179 (70%).

1-Hydroxy-7*H*-dibenzo[*de*,*g*]quinolone-7-one (**3**): Orange solid; mp 161–164 °C; IR (cm^−1^): 3628, 1647, 1635; UV (MeOH) λ_max_: 213, 242, 247, 253, 297, 378 nm; HRMS–ESI (*m*/*z*): [M + H]^+^ calcd for 248.0706; found, 248.0715; EIMS *m*/*z*: 221 (18%), 220 (98%), 192 (70%).

1,8-Dihydroxy-9-methoxy-7*H*-dibenzo[*de*,*g*]quinolone-7-one (**4**): Orange solid; mp 192–194 °C; IR (cm^−1^): 3600, 1667, 1615; UV (MeOH) λ_max_: 212, 261, 320, 333, 403 nm; HRMS–ESI (*m*/*z*): [M + H]^+^ calcd for 294.0761; found, 294.0770; EIMS *m*/*z*: 266 (100%), 248 (58%), 237 (43%), 223 (83%).

#### Larvicidal assay

For a preliminary test, stock solutions were prepared by dissolving 10 mg of each extract in 1 mL of acetone or absolute ethanol for the methanol fraction and then adding 99 mL of distilled water to make 100 mL (100 μg/mL). Each of the prepared solutions was divided into five portions of 20 mL in 30 mL vials. Five third-instar *Anopheles gambiae* larvae were introduced into each vial and fed with fish food. Mortality was recorded after 14, 48 and 72 hours. Control experiments were done by adding 1 mL of acetone into a 150 mL flask and then adding 99 mL of distilled water and applying the same procedure as mentioned above.

Extracts and isolated compound showing high larvicidal activity were tested at 10, 5, 2, and 1 ppm. This was performed by dissolving 5, 2.5, 1 and 0.5 mg of these organic materials in 1 mL of acetone and adding 499 mL of distilled water in a 500 mL beaker. Each of the prepared solutions was divided into five portions of 100 mL each in 250 mL beakers. Then, ten third-instar *A. gambiae* larvae were introduced into each beaker and fed with fish food. The mortality was recorded after 24, 48, and 72 hours. A regression equation obtained was used to determine the lethal concentration for 24, 48 and 72 hours.

## Supporting Information

File 1 NMR spectra of compounds.
