# Supplementary material for: Zanthoxoaporphines A–C: Three new larvicidal dibenzo[de,g]quinolin-7-one alkaloids from Zanthoxylum paracanthum (Rutaceae)
Source: Beilstein J Org Chem. 2013 Feb 27;9:447–52. doi: 10.3762/bjoc.9.47 (PMC3596013; doi:10.3762/bjoc.9.47)
Supplement: File 1 — NMR spectra of compounds. [file Beilstein_J_Org_Chem-09-447-s001.pdf]

Supporting Information

for

Zanthoxoaporphines A–C: Three new larvicidal dibenzo[*de,g*]quinolin-7-one alkaloids from *Zanthoxylum paracanthum* (Rutaceae)

Fidelis N. Samita[1,2], Louis P. Sandjo[3*], Isaiah O. Ndiege[2‡*], Ahmed Hassanali[2], Wilber Lwande[4]

Address: [1]Department of Chemistry, Maseno University, P.O. Private Bag Maseno, Kenya, [2]Department of Chemistry, Kenyatta University, P.O. Box 43844, Nairobi 00100, Kenya, [3]Department of Organic chemistry, University of Yaoundé 1, P.O. Box 812 Yaoundé Cameroon and [4]Behavioural and Chemical Ecology Department, International Centre for Insect Physiology and Ecology, P.O. Box 30772, Nairobi 00100, Kenya

Email: Louis P. Sandjo - plsandjo@yahoo.fr, Isaiah O. Ndiege - indiege@yahoo.com

‡Tel. +254-721818654

*Corresponding author

NMR spectra of compounds

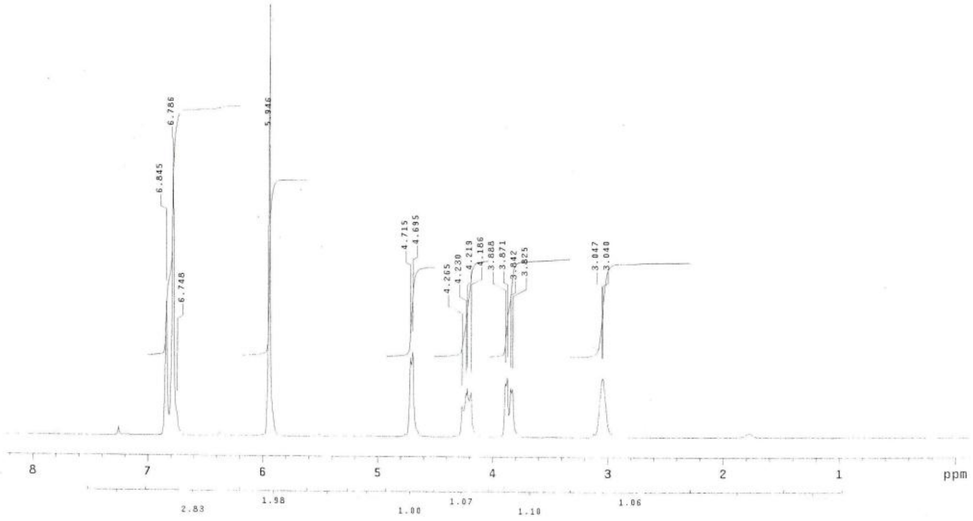

Figure 1: ^{1}H NMR spectrum of compound **1** (300 MHz, $CDCl_3$).

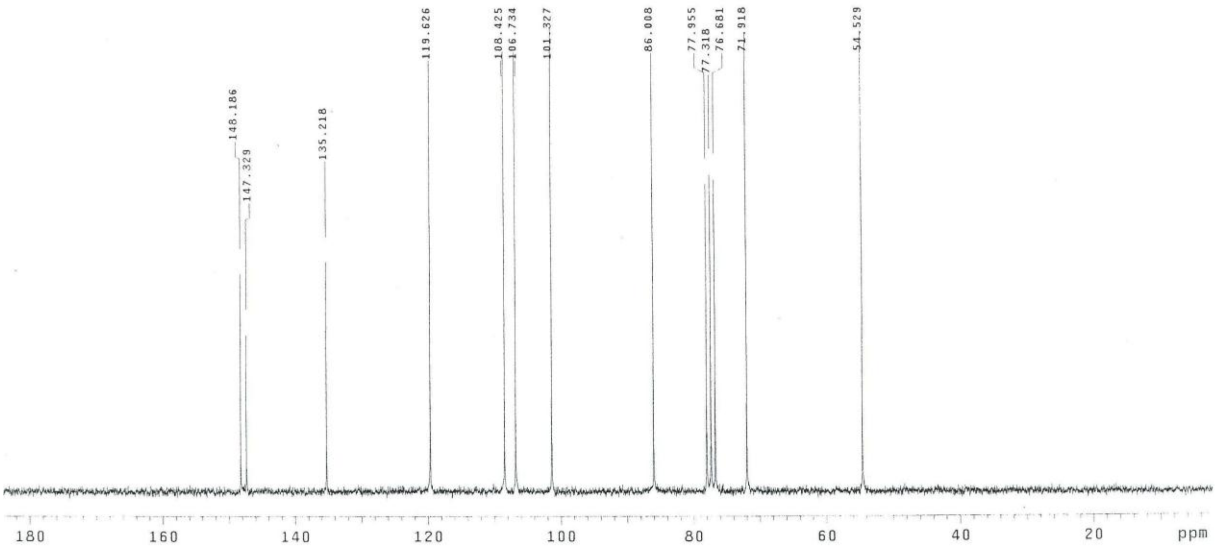

Figure 2: ^{13}C NMR spectrum of compound **1** (75 MHz, $CDCl_3$).

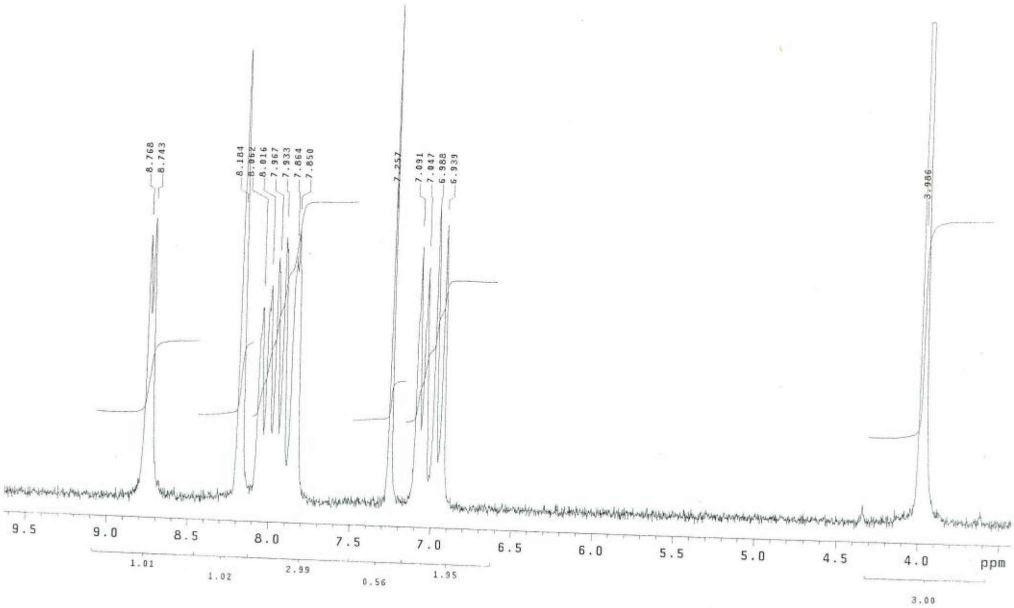

Figure 3: ^{1}H NMR spectrum of compound **2** (300 MHz, $CDCl_3$).

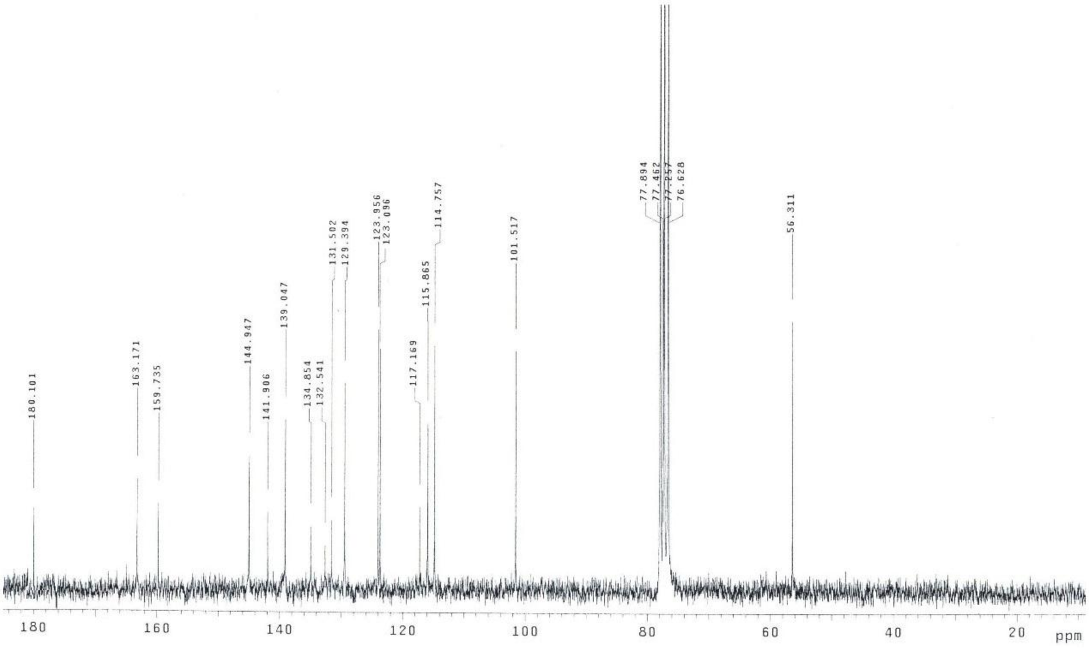

Figure 4: ^{13}C NMR spectrum of compound **2** (75 MHz, $CDCl_3$).

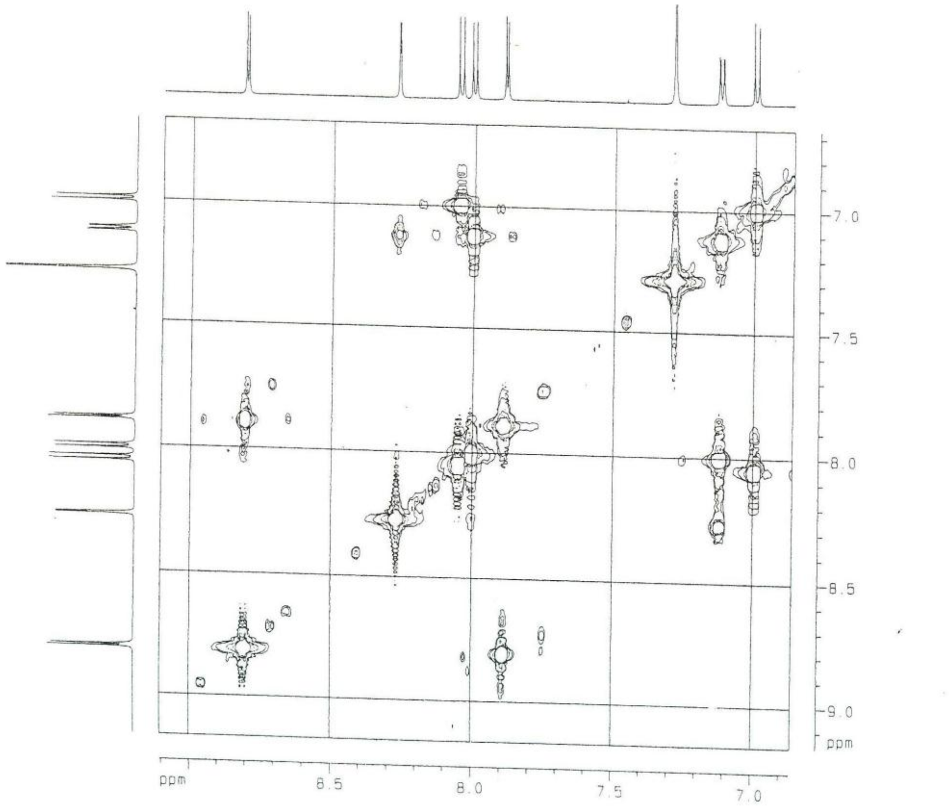

Figure 5: COSY spectrum of compound **2** (600 MHz, $CDCl_3$).

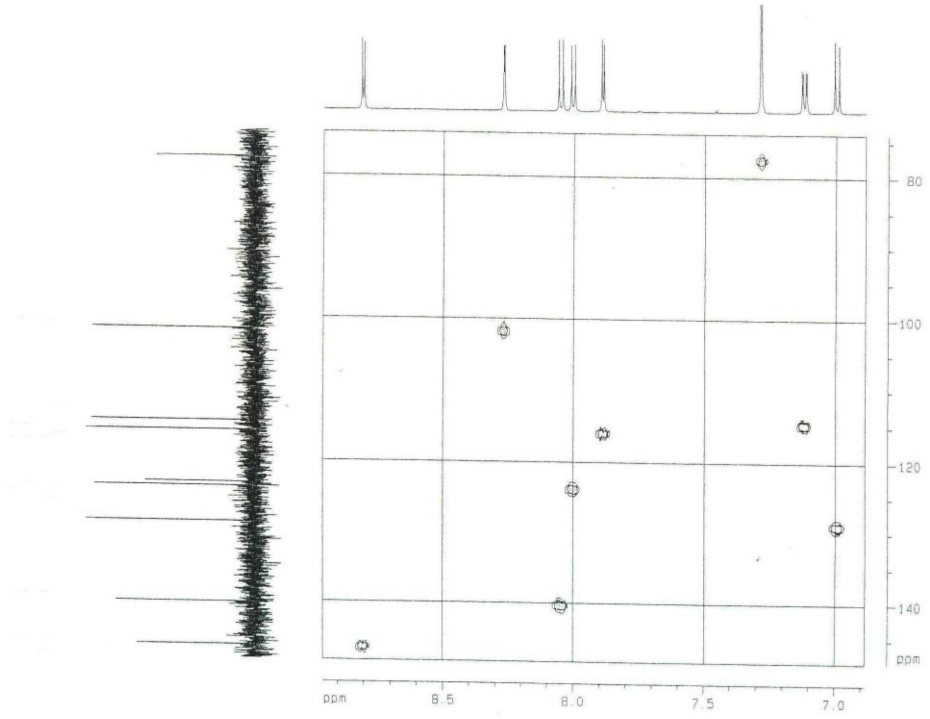

Figure 6: HMQC spectrum of compound **2** (600 MHz, $CDCl_3$).

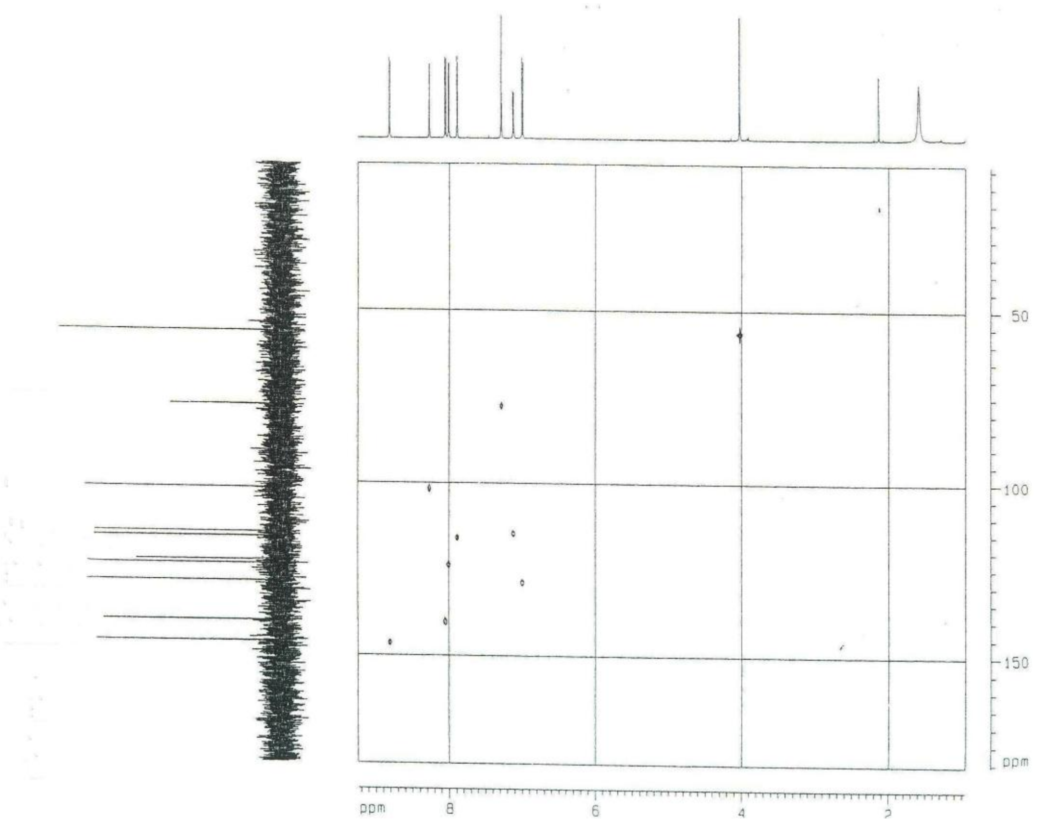

Figure 7: HMQC spectrum of compound **2** (600 MHz, $CDCl_3$).

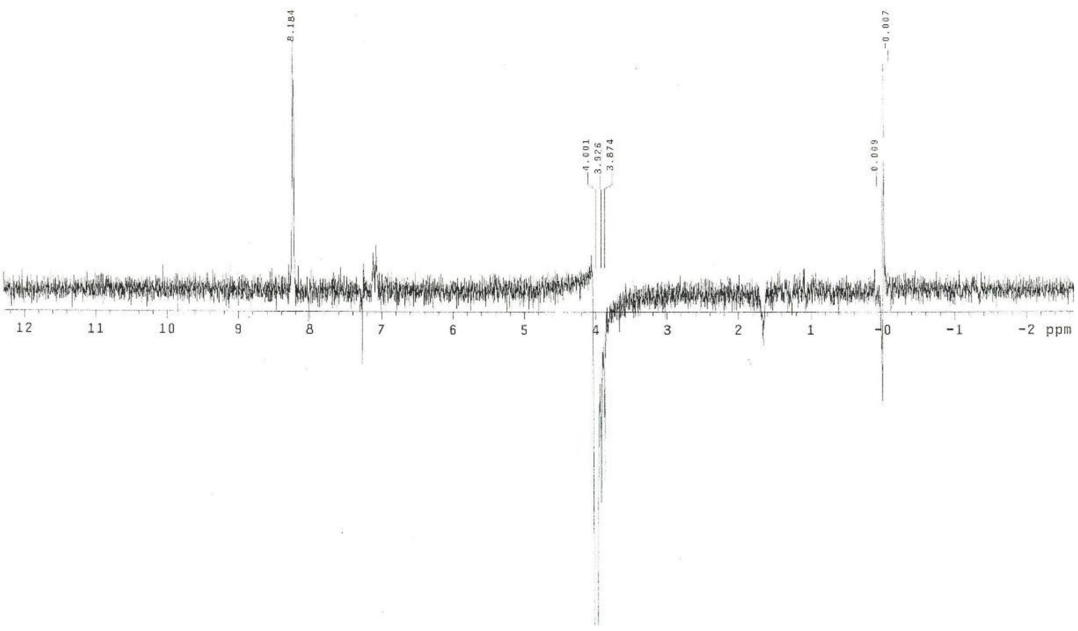

Figure 8: NOE spectrum of compound **2**.

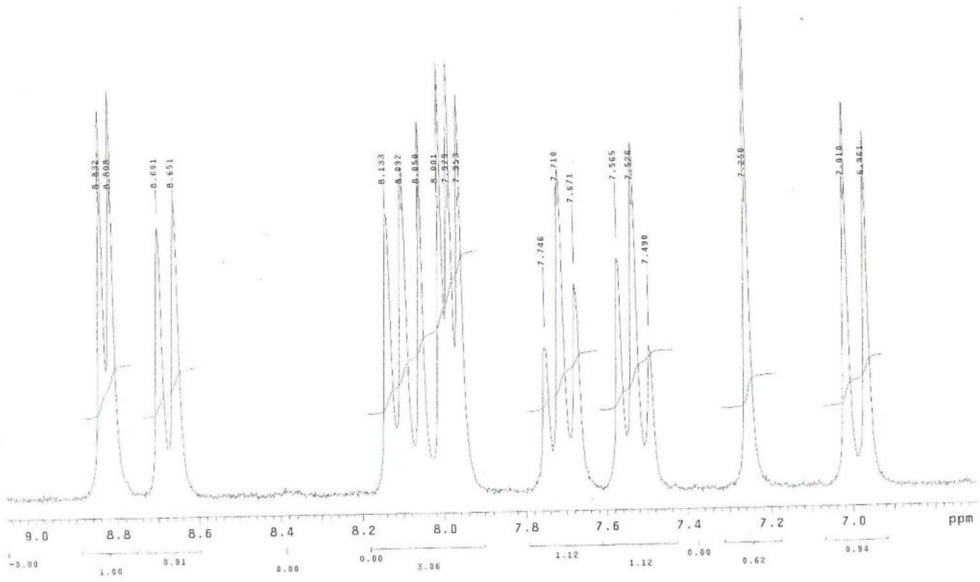

Figure 9: ^{1}H NMR spectrum of compound **3** (300 MHz, $CDCl_3$).

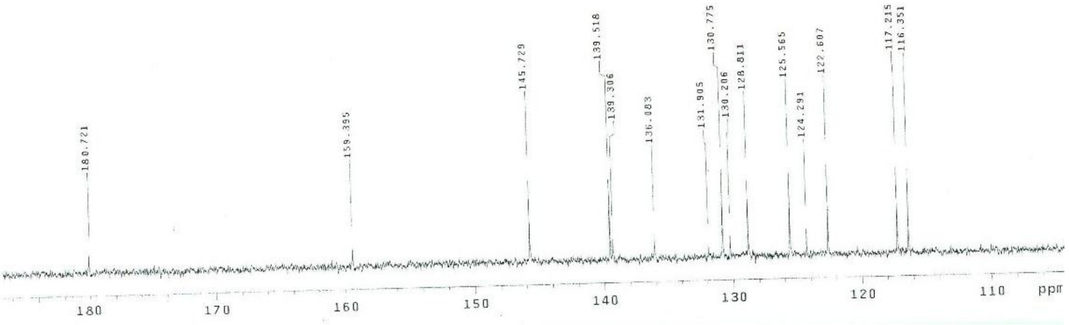

Figure 10: ^{13}C NMR spectrum of compound **3** (75 MHz, $CDCl_3$).

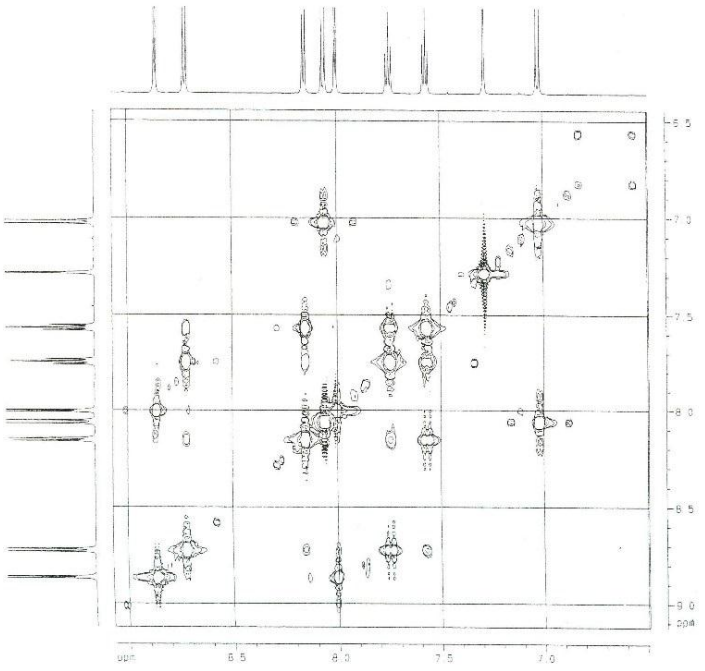

Figure 11: COSY spectrum of compound **3** (600 MHz, $CDCl_3$).

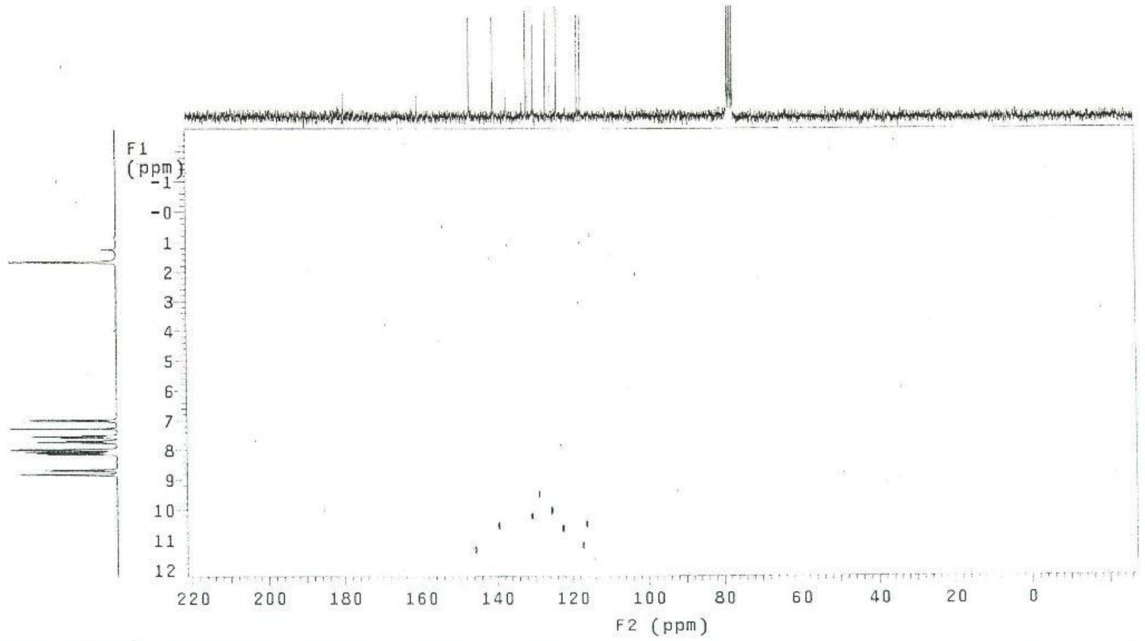

Figure 12: HETCOR spectrum of compound **3**.

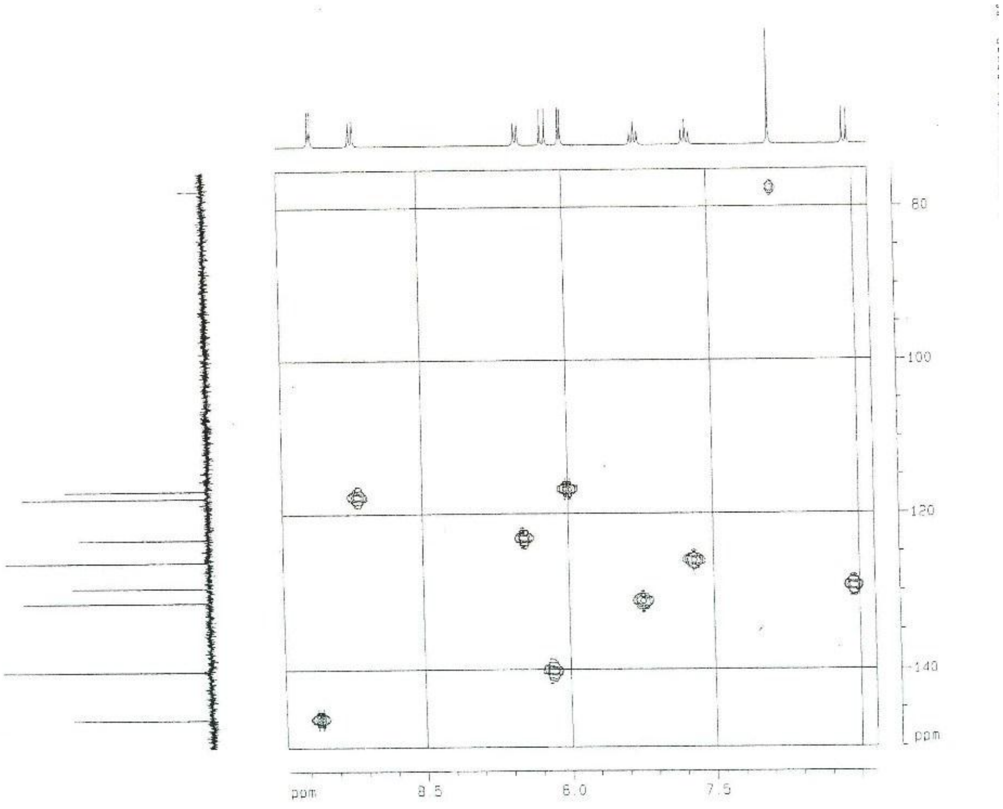

Figure 13: HMQC spectrum of compound **3**.

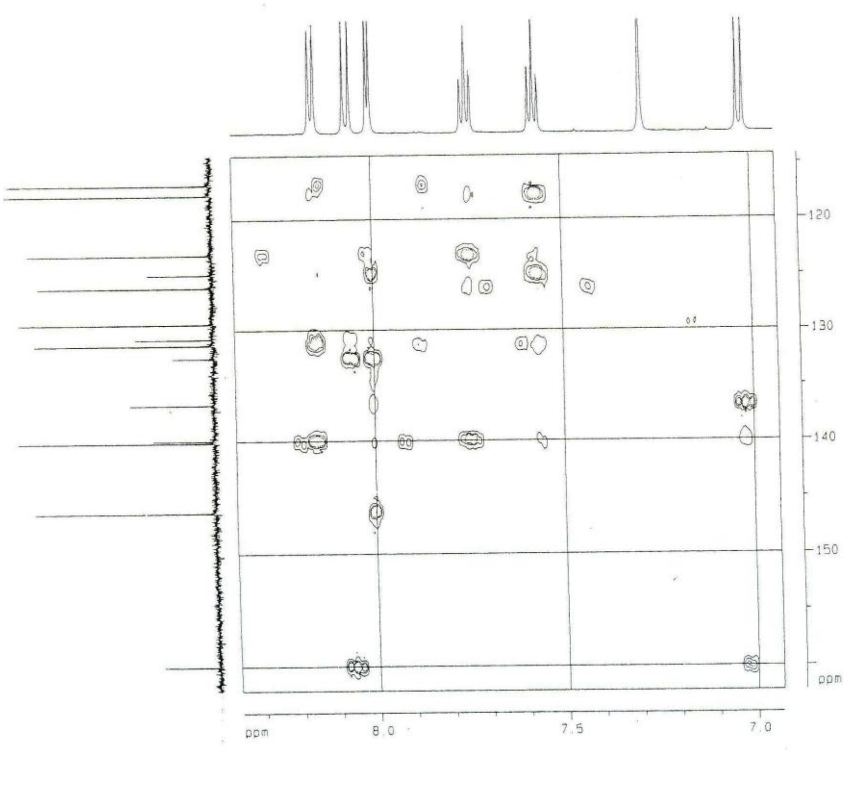

Figure 14: HMBC spectrum of compound **3**.

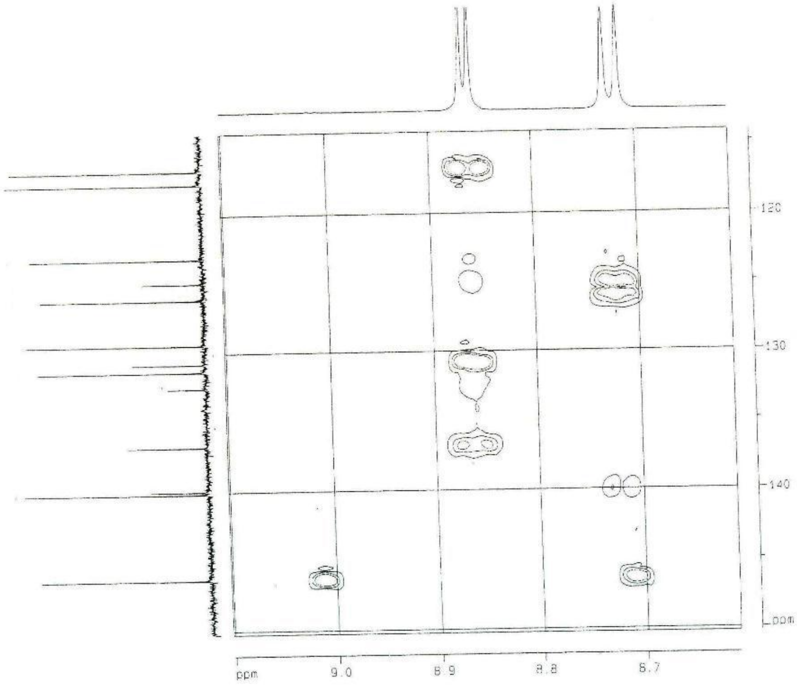

Figure 15: HMBC spectrum of compound **3**.

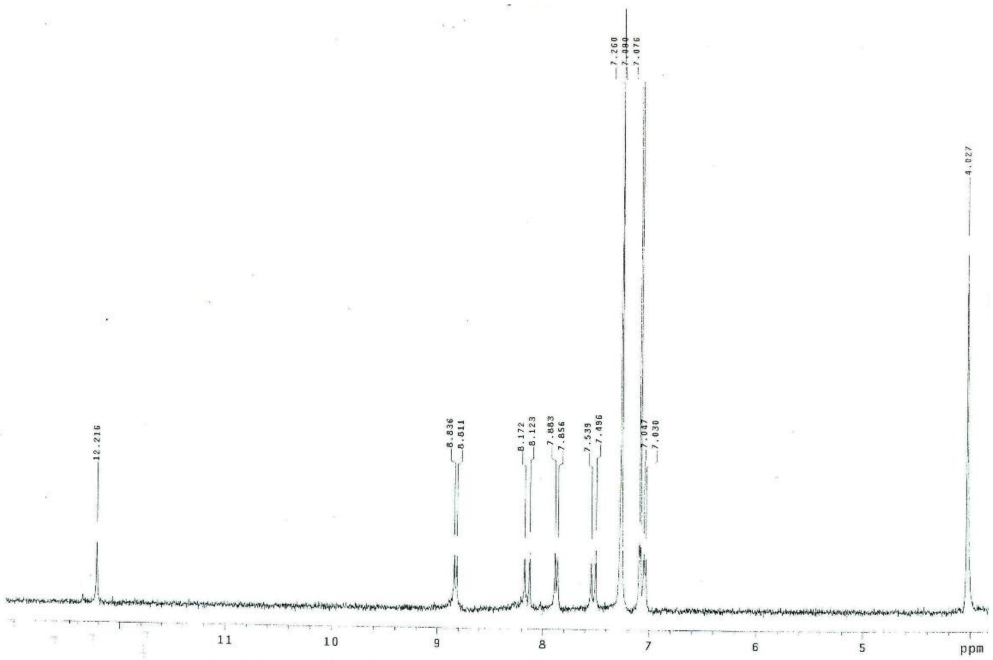

Figure 16: ^{1}H NMR spectrum of compound **4** (300 MHz, $CDCl_3$).

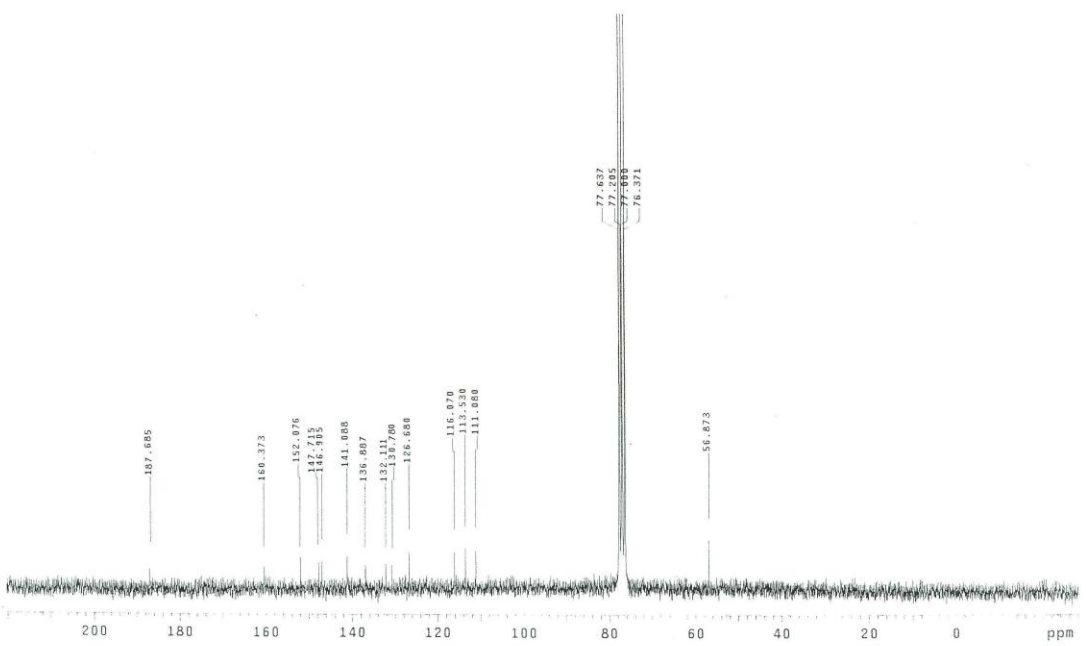

Figure 17: ^{13}C NMR spectrum of compound **4** (75 MHz, $CDCl_3$).

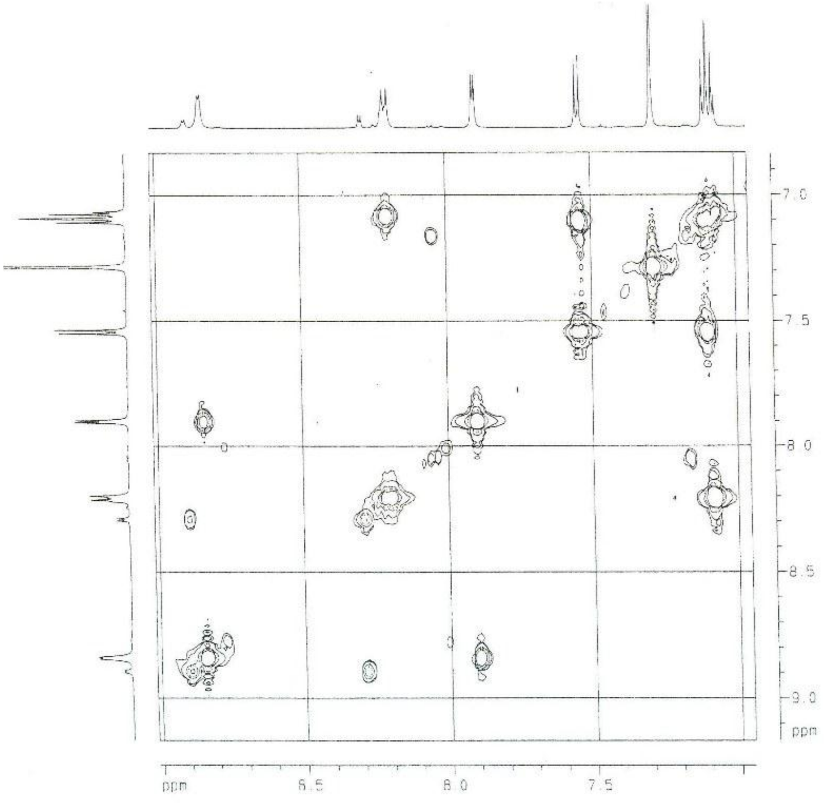

Figure 18: COSY spectrum of compound **4** (600 MHz, $CDCl_3$).

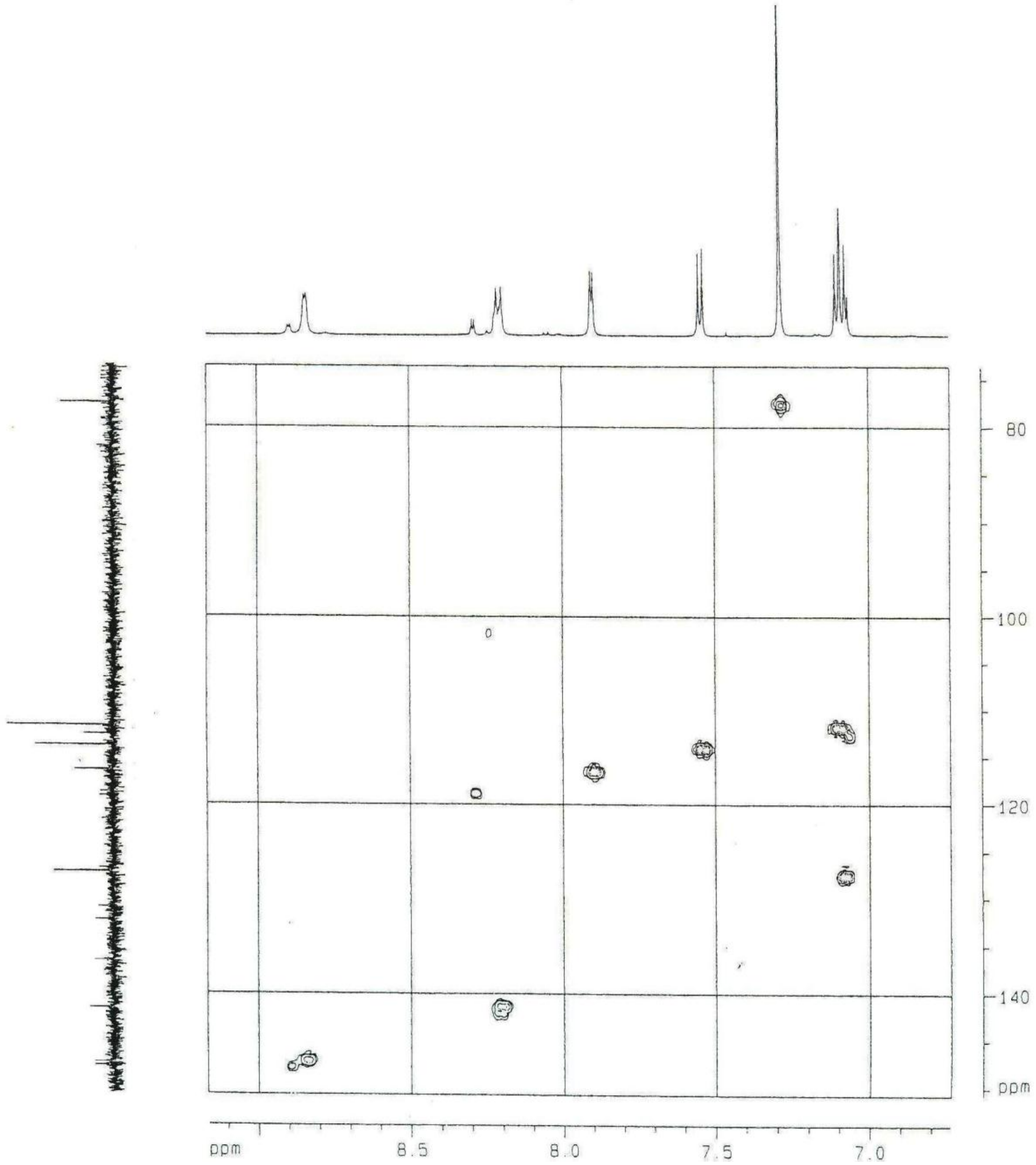

Figure 19: HMQC spectrum of compound **4** (600 MHz, $CDCl_3$).

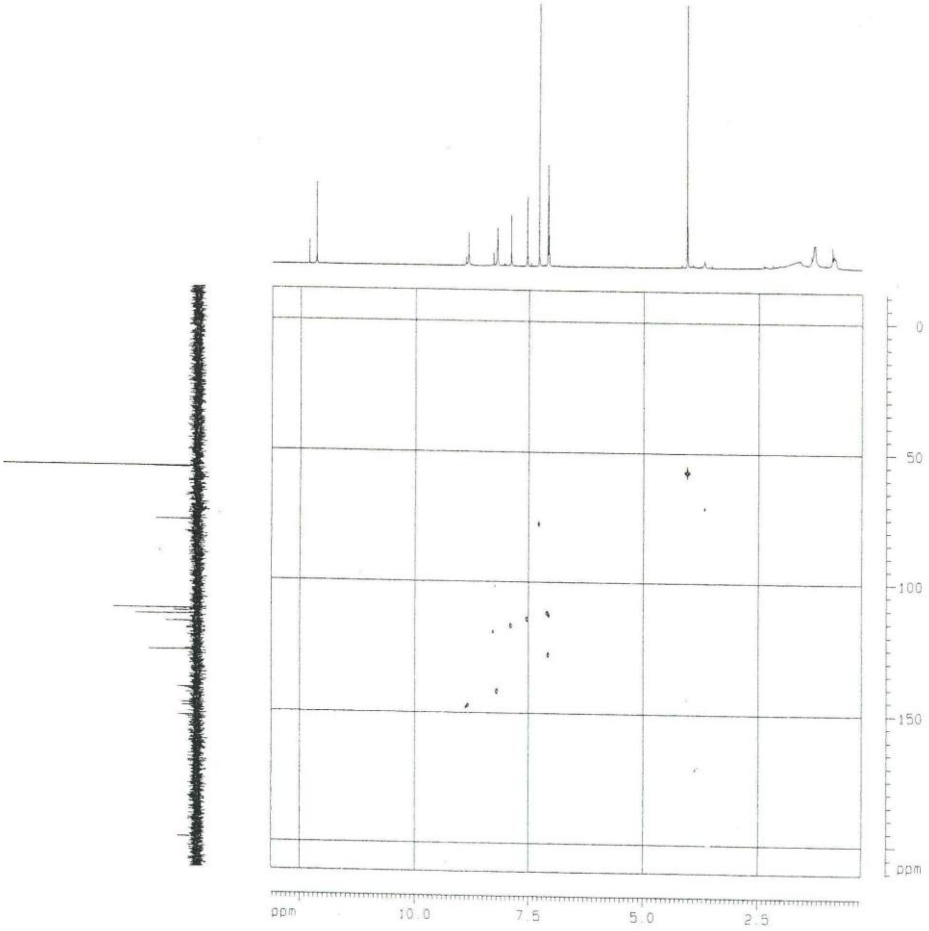

Figure 20: HMQC spectrum of compound **4** (600 MHz, $CDCl_3$).

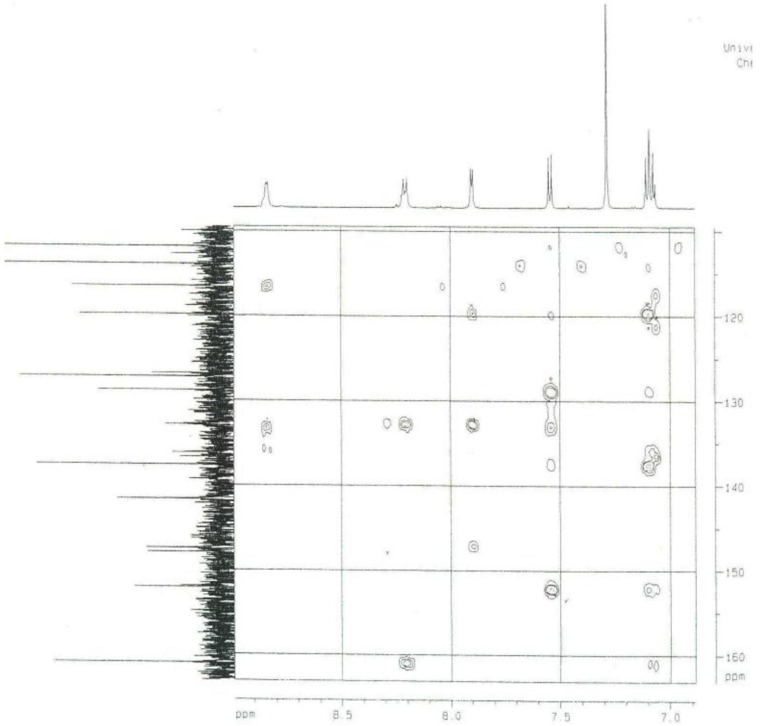

Figure 21: HMQC spectrum of compound **4** (600 MHz, $CDCl_3$).

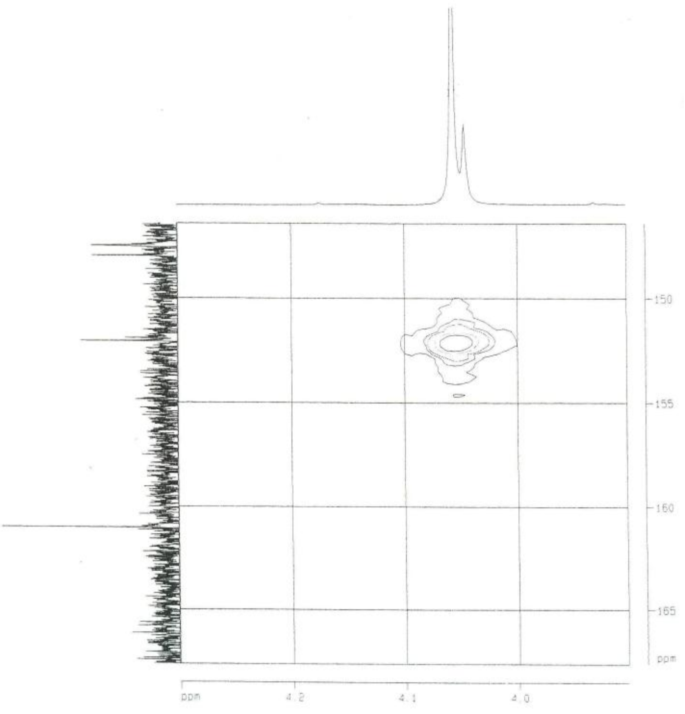

Figure 22: HMQC spectrum of compound **4** (600 MHz, $CDCl_3$).

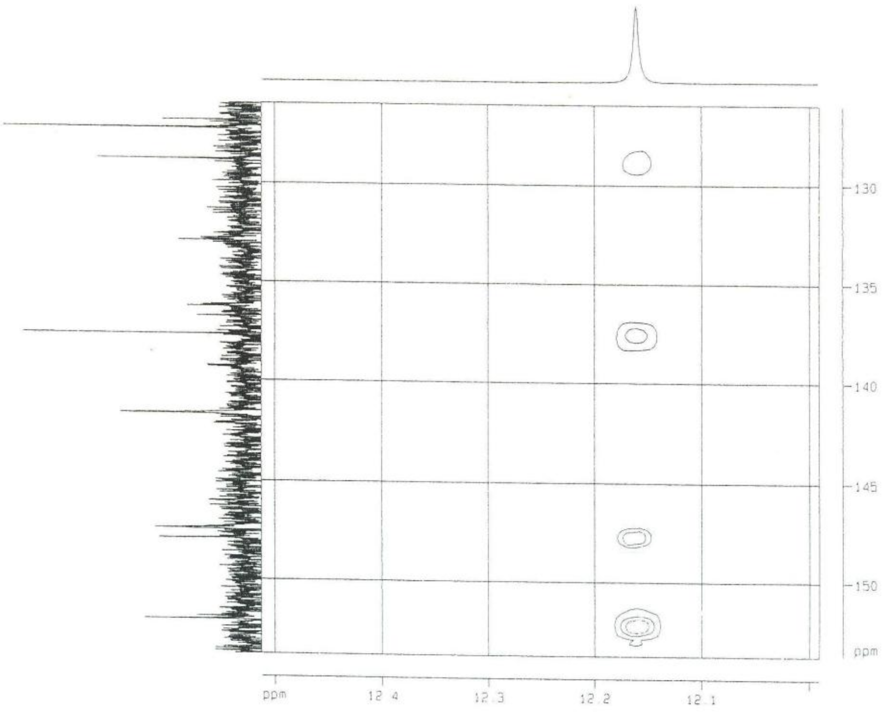

Figure 23: HMQC spectrum of compound **4** (600 MHz, $CDCl_3$).
